# Strategies for the use of *Ginkgo biloba* extract, EGb 761^®^, in the treatment and management of mild cognitive impairment in Asia: Expert consensus

**DOI:** 10.1111/cns.13536

**Published:** 2020-12-22

**Authors:** Nagaendran Kandiah, Yee Fai Chan, Christopher Chen, Darwin Dasig, Jacqueline Dominguez, Seol‐Heui Han, Jianping Jia, SangYun Kim, Panita Limpawattana, Li‐Ling Ng, Dinh Toan Nguyen, Paulus Anam Ong, Encarnita Raya‐Ampil, Nor'izzati Saedon, Vorapun Senanarong, Siti Setiati, Harjot Singh, Chuthamanee Suthisisang, Tong Mai Trang, Yuda Turana, Narayanaswamy Venketasubramanian, Fee Mann Yong, Yong Chul Youn, Ralf Ihl

**Affiliations:** ^1^ National Neuroscience Institute Singapore Singapore; ^2^ Duke‐NUS Singapore Singapore; ^3^ Lee Kong Chian‐Imperial College Singapore Singapore; ^4^ Hospital Kuala Lumpur Kuala Lumpur Malaysia; ^5^ Departments of Pharmacology and Psychological Medicine Yong Loo Lin School of Medicine Memory Aging and Cognition Centre National University of Singapore Singapore Singapore; ^6^ Makati Medical Center Manila Philippines; ^7^ St Luke's Medical Center Manila Philippines; ^8^ Konkuk University Medical Center Seoul Korea; ^9^ Xuanwu Hospital Capital Medical University Beijing China; ^10^ Department of Neurology Seoul National University College of Medicine and Seoul National University Bundang Hospital Seoul Korea; ^11^ Srinakarind Hospital Faculty of Medicine Khon Kaen University Khon Kaen Thailand; ^12^ Changi General Hospital Singapore Singapore; ^13^ Department of Internal Medicine University of Medicine and Pharmacy Hue University Hue City Vietnam; ^14^ Hasan Sadikin General Hospital Jawa Barat Indonesia; ^15^ University of Santo Tomas Metro Manila Philippines; ^16^ Department of Medicine University of Malaya Kuala Lumpur Malaysia; ^17^ Faculty of Medicine Siriraj Hospital Mahidol University Bangkok Thailand; ^18^ Department of Internal Medicine Cipto Mangunkusumo Hospital Jakarta Indonesia; ^19^ Dr Harjot Singh's Neuropsychiatry Centre and Hospital Amritsar India; ^20^ Faculty of Pharmacy Mahidol University Bangkok Thailand; ^21^ Department of Neurology University Medical Center Ho Chi Minh City Vietnam; ^22^ School of Medicine and Health Science Atma Jaya Catholic University of Indonesia Jakarta Indonesia; ^23^ Raffles Neuroscience Centre Raffles Hospital Singapore Singapore; ^24^ Subang Jaya Medical Centre Selangor Malaysia; ^25^ Chung‐Ang University Medical Center Seoul Korea; ^26^ Alexian Hospital Krefeld Germany

**Keywords:** cerebrovascular disease, dementia, EGb 761^®^, *Ginkgo biloba*, mild cognitive impairment, neuropsychiatric symptoms

## Abstract

**Background:**

Mild cognitive impairment (MCI) is a neurocognitive state between normal cognitive aging and dementia, with evidence of neuropsychological changes but insufficient functional decline to warrant a diagnosis of dementia. Individuals with MCI are at increased risk for progression to dementia; and an appreciable proportion display neuropsychiatric symptoms (NPS), also a known risk factor for dementia. Cerebrovascular disease (CVD) is thought to be an underdiagnosed contributor to MCI/dementia. The Ginkgo biloba extract, EGb 761^®^, is increasingly being used for the symptomatic treatment of cognitive disorders with/without CVD, due to its known neuroprotective effects and cerebrovascular benefits.

**Aims:**

To present consensus opinion from the ASian Clinical Expert group on Neurocognitive Disorders (ASCEND) regarding the role of EGb 761^®^ in MCI.

**Materials & Methods:**

The ASCEND Group reconvened in September 2019 to present and critically assess the current evidence on the general management of MCI, including the efficacy and safety of EGb 761^®^ as a treatment option.

**Results:**

EGb 761^®^ has demonstrated symptomatic improvement in at least four randomized trials, in terms of cognitive performance, memory, recall and recognition, attention and concentration, anxiety, and NPS. There is also evidence that EGb 761^®^ may help delay progression from MCI to dementia in some individuals.

**Discussion:**

EGb 761^®^ is currently recommended in multiple guidelines for the symptomatic treatment of MCI. Due to its beneficial effects on cerebrovascular blood flow, it is reasonable to expect that EGb 761^®^ may benefit MCI patients with underlying CVD.

**Conclusion:**

As an expert group, we suggest it is clinically appropriate to incorporate EGb 761^®^ as part of the multidomain intervention for MCI.

## Graphical abstract

EGb 761^®^ has demonstrated improvement in mild cognitive impairment (MCI) symptoms in at least four randomized trails, and is currently recommended for MCI in multiple clinical guidelines. The ASian clinical expert group on Neurocognitive Disorders (ASCEND) suggests that it is clinically appropriate to incorporate EGb 761^®^ as part of the multidomain intervention for MCI, including cases with underlying CVD.

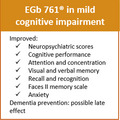

## 1 INTRODUCTION

The global burden of cognitive disorders is rapidly becoming a major chronic health issue worldwide. Currently, approximately 50 million individuals are living with dementia, and this number is expected to approximately double in the next two decades, with much of the increase likely to be attributable to low‐to‐middle income countries, particularly within Asia.^1^ As well as dementia sufferers, a great many more elderly individuals have less pronounced, but nonetheless distressing, signs of cognitive decline and memory loss.^2^, ^3^

Mild cognitive impairment (MCI) is a clinically defined neurocognitive state between normal cognitive aging and dementia, with evidence of neuropsychological changes but without sufficient functional decline to warrant a diagnosis of dementia.^4^, ^5^ Individuals with MCI are at increased risk for progression to dementia,^3^, ^6^, ^7^, ^8^, ^9^ but only a small proportion of these patients are likely to present for diagnosis and treatment. Hence, there is a need to raise greater awareness of this syndrome and its management.^4^

The current prevalence of MCI is difficult to establish due to a lack of standardized diagnostic criteria and assessment procedures.^2^, ^10^ Estimates vary depending on the country, the diagnostic criteria used, and the age of the study population.^10^, ^11^ MCI prevalence increases with advancing cohort age; it is estimated that 6.7% of individuals aged 60–64 have MCI, increasing to 8.4% for ages 65–69, 10.1% for ages 70–74, 14.8% for ages 75–79, and 25.2% for ages 80–84.^3^ Depending on the arbitrary criteria used to define MCI, the global prevalence of MCI is reported to be approximately threefold higher than the global prevalence of dementia (5%–7% in all individuals over 60 years of age).^12^

Asian data suggest an overall MCI standardized prevalence of around 5%–20%, depending on the country, the age of the cohort, and the criteria applied.^11^, ^13^, ^14^, ^15^, ^16^, ^17^, ^18^ A recent meta‐analysis of 48 studies from China reported a pooled prevalence of 14.7% in individuals aged ≥60 years.^18^ Overall, these MCI data are approximately double the reported prevalence of dementia across various Asian countries (2%–13%).^19^, ^20^

### 1.1 Definition of MCI

Making a distinction between normal cognitive aging and MCI remains a clinical challenge. Following subjective memory complaints, a diagnosis requires the use of validated tools for cognitive testing and functional assessment.^21^ Historically, the criteria for MCI have included subjective complaints and objective cognitive impairments in domains such as memory, executive functioning, attention, language, and visuospatial skills, but without impairment in function or activities of daily living (ADL).^2^, ^6^, ^22^ However, more recently, it is acknowledged that subtle deficits in more complex instrumental ADL may indeed be present in patients with MCI.^6^, ^23^

Two broad subtypes of MCI (amnestic and nonamnestic) are recognized, based on whether or not memory impairment is present. Depending on whether more than one domain is impaired, both subtypes can be further categorized as either single domain or multidomain.^24^

### 1.2 Neuropsychiatric symptoms in MCI

Neuropsychiatric symptoms (NPS), also known as “noncognitive behavioral and psychological symptoms of dementia,” may include depression, anxiety, irritability, apathy, and changes in personality or usual behaviors.^25^, ^26^, ^27^ NPS are observed across the severity spectrum of dementia, and are common in patients with MCI.^25^, ^26^ Reports are variable, but between 35% and 85% of MCI patients may display NPS.^25^, ^26^, ^27^, ^28^ The presence of NPS in MCI increases the caregiver's burden^29^ and is also associated with an increased risk of incident dementia.^30^, ^31^, ^32^

### 1.3 Pathophysiology of MCI

Pathologic evidence of Alzheimer's disease (AD) has been observed in individuals with memory impairment but no clinical manifestation of AD, indicating that the degenerative process may begin years before clinical manifestation.^33^, ^34^, ^35^ The pathophysiology of age‐associated memory disorders appears to be multifactorial. Across the spectrum of age‐related memory and cognitive disorders, neurodegenerative changes (ie, synaptic deficits and neuronal loss) and histopathological alterations (ie, increased production of β‐amyloid leading to extracellular amyloid‐containing plaques, and formation of intracellular hyperphosphorylated tau‐protein tangles) have been observed.^36^ Impaired cerebral glucose metabolism in memory‐related brain regions has also been reported.^37^ There is increasing evidence that mitochondrial dysfunction, in terms of reduced mitochondrial enzyme function and increased oxidative stress, is a major pathomechanistic contributor to these findings.^36^

As well as neurodegenerative brain changes, vascular pathologies such as small vessel disease (eg, ischemic white matter changes, multiple lacunar infarcts), large vessel disease (eg, multiple infarcts, single strategically placed infarcts), or hemorrhage (eg, multiple microbleeds) have been frequently reported in dementia, and may be evident before overt clinical symptoms arise.^38^, ^39^, ^40^ Cerebrovascular disease (CVD) is thought to be underdiagnosed^19^ and underestimated as a potential cause of MCI.^4^ In this context, there is some evidence for a relatively higher prevalence of CVD among Asian patients with MCI and dementia, compared with Western populations.^41^, ^42^ In Asia, CVD is reported to account for approximately 4% of MCI cases^17^ and approximately 20% of dementia cases,^19^ with variation between and within countries.^16^, ^17^, ^19^

### 1.4 Risk of progression from MCI to dementia

Because MCI is a known risk factor for, and an early manifestation of, dementia and other neurodegenerative disorders,^3^, ^6^, ^7^, ^8^, ^9^ early, accurate diagnosis of MCI represents an important opportunity for therapeutic intervention.^2^, ^3^, ^22^

The rate of progression to dementia has been estimated at between 10% and 19% per year in individuals with MCI, compared with 1%–2% in the general population.^3^, ^22^, ^43^, ^44^, ^45^ Consistent with this, a large systematic review reported MCI‐to‐dementia progression rates of 10%–36% over 2 years,^43^ while the German AgeCoDe study reported that approximately 40% of patients aged ≥75 years with amnestic MCI progressed to dementia over 3 years of follow‐up.^46^ In a large, prospective Australian study, over 30% of individuals with MCI (mean age, 76 years) transitioned to AD within an 18‐month follow‐up period.^47^

Although between 15% and 50% of patients may revert to normal cognition after a diagnosis of MCI,^3^, ^46^, ^48^ these individuals remain at significantly higher risk of MCI re‐diagnosis and ultimate progression to dementia.^3^, ^48^

It is important to identify MCI patients who are at higher risk for dementia, to ensure early intervention. As well as the presence of NPS,^30^, ^31^, ^32^ risk factors such as age,^45^ diabetes mellitus,^30^ baseline memory impairment,^33^, ^49^ sharp decline in functional ability,^49^ impaired executive performance,^33^ and instrumental ADL deficits^23^ may increase the likelihood of further cognitive decline. A number of biomarkers such as hippocampal volume, medial temporal lobe cortical thickness, and indicators of CVD such as infarcts, white matter hyperintensities, lacunes, and microbleeds, have all shown an association with progression to dementia.^33^, ^45^, ^49^ The knowledge that CVD markers may serve as prognostic indicators for further cognitive decline underlines the importance of managing vascular risk factors to slow the progression of MCI to dementia.^41^, ^50^, ^51^

### 1.5 Ginkgo biloba extract, EGb 761^®^

EGb 761^®^ is a dry extract from the leaves of the *Ginkgo biloba* plant, derived through a proprietary process. Depending on local regulatory frameworks across Asia, EGb 761^®^ may be either classified as a drug, a supplement, or a phytopharmaceutical. This extract has increasingly been used over the past two decades for the symptomatic treatment of cognitive disorders, including AD with or without CVD.^52^, ^53^, ^54^, ^55^, ^56^, ^57^ A recent comprehensive review reported that EGb 761^®^ improved cognitive performance across the spectrum of age‐associated cognitive disorders, from age‐associated memory complaints through to AD and vascular dementia (VaD).^36^

EGb 761^®^ exhibits various beneficial properties, although its mechanism of action in cognitive disorders is not yet fully understood. Preclinical evidence suggests that EGb 761^®^ has profound effects on mitochondrial function via several mechanisms, including potent antioxidant activity.^36^ This agent has been shown to reduce oxidative cell damage through reducing mitochondrial production of reactive oxygen species, due to its high levels of antioxidants (flavonoids and terpenoids).^36^, ^58^ EGb 761^®^ also protects neurons from amyloid‐beta (Aβ)‐induced toxicity through inhibiting the formation of Aβ oligomers,^36^, ^59^ and affects the insulin receptor by influencing acetylcholine reduction.^36^, ^60^, ^61^, ^62^ Secondary to these important properties, EGb 761^®^ appears to have profound effects on neuronal function, neuroplasticity, neuroregeneration, and neuroinflammation,^36^, ^60^ and positively influences synaptic plasticity and brain functions that require high amounts of cellular energy.^36^ Additionally, evidence suggests that EGb 761^®^ increases cerebral blood flow and brain perfusion by decreasing cerebral blood viscosity, and protects cerebral blood vessels against processes involved in atherosclerosis.^63^, ^64^, ^65^, ^66^ EGb 761^®^ has also been shown to increase dopamine levels in the prefrontal cortex.^67^

Thus, the known mechanisms of action of EGb 761^®^ provide strong rationale for its use in age‐related cognitive disorders.^36^, ^58^, ^60^, ^61^, ^62^

### 1.6 Rationale for this article

The ASian Clinical Expert group on Neurocognitive Disorders (ASCEND) consists of more than twenty members, primarily from the Asian region. The group first convened in 2017, and consists of experts from various specialties, including neurology, geriatrics, psychiatry, and pharmacy.

Following the first meeting, the ASCEND group published a set of evidence‐based regional consensus recommendations on the use of EGb 761^®^ in the treatment of dementia and MCI with or without CVD.^58^ Among the core consensus statements published, the ASCEND Expert Group recommended the use of EGb 761^®^ 240 mg/day as part of the treatment approach for AD (±CVD), VaD, and mixed dementia, with or without NPS. It was further agreed that EGb 761^®^ might be considered for use in patients with MCI (Class IIB recommendation; Level A evidence).^58^ These recommendations are consistent with a number of current guidelines and consensus documents from around the world, including Asia, that support the use of EGb 761^®^ for the treatment of MCI symptoms.^58^, ^68^, ^69^, ^70^, ^71^, ^72^

The purpose of this article is to expand upon these recommendations, with a specific focus on the role of EGb 761^®^ in the management of MCI. The potential role of EGb 761^®^ in slowing cognitive decline is also discussed.

## 2 METHODS

The ASCEND Group reconvened in September 2019 to present and critically assess the current evidence on the general management of MCI, particularly with respect to the clinical efficacy and safety of EGb 761^®^ as a treatment option for MCI. The preplanned output from the meeting was the construction of another regional consensus document to assist Asian countries in formulating strategies to improve the treatment and management of MCI.

Before the meeting, a survey was circulated to all ASCEND members to gather their expert opinions regarding MCI diagnosis and management in their respective clinical practices. In addition, a literature search was performed to identify primarily English language articles relevant to the use of EGb 761^®^ in MCI, using MeSH terms and other keywords, including: EGb 761, *Ginkgo biloba* extract, mild cognitive impairment, MCI, cognitive dysfunction, memory disorders, subjective memory loss, dementia, neuropsychiatric symptoms, cerebrovascular disorders, and pathophysiology.

During the meeting, two didactic presentations from key opinion leaders summarized the diagnosis and burden of MCI, and the current evidence relating to both pharmacological and nonpharmacological management approaches, in terms of symptomatic improvement and stabilization of progression. The expert group used this information as the basis for discussion of pertinent topics in the context of their clinical expertise. A series of proposed consensus statements were subsequently formulated and discussed among the expert group via email, as part of the construction of the present article. All members reviewed the final manuscript and reached consensus on each statement presented herein.

## 3 RESULTS

### 3.1 General principles of MCI management

Ideally, goals of treatment for dementia—and indeed for MCI—are improvement of cognitive function and psychological and behavioral symptoms, stabilization or slowing of disease progression, improvement of quality of life (QoL), and alleviation of caregiver burden.^19^ As mixed pathology is common in MCI, a multidomain management approach that benefits both neurodegenerative and vascular pathology is a rational strategy.^73^

Based on the literature and clinical experience, the ASCEND Expert Group recommends that clinicians first assess for reversible causes of subjective cognitive impairment (SCI), such as depression, medications, alcohol use disorders, or hearing loss, and manage these appropriately.^3^, ^74^ Validated neurocognitive and functional assessment tools should be used for the diagnosis and monitoring of MCI in individuals with persistent memory complaints.^3^ Other investigations may include a blood panel and brain imaging.^21^, ^75^

Second, it is important to mitigate any vascular and lifestyle risk factors. This approach has recently proven successful as an early intervention for dementia prevention and delay.^76^, ^77^ To this end, recent recommendations from the WHO (2019)^74^ and AAN^3^ include physical exercise, nutritional and weight loss interventions, tobacco cessation, and management of hypertension, diabetes mellitus, and dyslipidemia. The Finnish Geriatric Intervention Study to Prevent Cognitive Impairment and Disability (FINGER) study was a 2‐year randomized trial of multidomain intervention in patients aged 60–77 years with cognition at or below the normative mean (although, not all patients had MCI, and the inclusion/exclusion criteria would have excluded some cases of MCI). The intervention cohort, which received interventions including good nutrition, physical exercise, vascular risk factor monitoring, and cognitive training, showed significant improvement in neuropsychological test battery scores per year, compared with a control cohort receiving regular health advice alone.^76^

Other interventions which may contribute to cognitive function include positive social activity^74^, ^78^, ^79^, ^80^ and sleep management.^81^, ^82^

### 3.2 Pharmacological interventions

Symptomatic pharmacological treatment options for MCI are limited; at present, there are no FDA‐approved treatments indicated specifically for MCI.^3^ While acetylcholinesterase inhibitors (AChEIs; eg, donepezil, rivastigmine, and galantamine) are used first‐line in AD and AD+CVD in Asia,^19^ these have not shown robust ability to improve MCI symptoms in well‐controlled randomized trials.^83^, ^84^, ^85^, ^86^ Thus, the AAN guidelines for MCI state that clinicians may choose *not* to offer cholinesterase inhibitors (Level B evidence), and, if offered, they should first discuss with patients the lack of evidence (Level A).^3^

A number of other off‐label treatments have been trialled.^87^ Among them, EGb 761^®^ is increasingly being used in the treatment of cognitive disorders,^53^, ^56^, ^57^ and the evidence supporting its efficacy in individuals with MCI is growing.

### 3.3 Efficacy of EGb 761^®^ in MCI

Based on evidence from randomized trials^52^, ^54^, ^55^ and meta‐analyses,^88^, ^89^, ^90^, ^91^ our first ASCEND consensus statement concluded that EGb 761^®^ has an important role in current best practice for the symptomatic treatment of AD (±CVD) and VaD.^58^ Further, based on clinical trial data^92^, ^93^, ^94^, ^95^ and pathomechanistic reasoning, we concluded that EGb 761^®^ may also be considered for use in patients with MCI, including those with evidence of CVD (class of recommendation, IIB; level of evidence, A).^58^

EGb 761^®^ is currently the only pharmacological agent recommended for the symptomatic treatment of MCI in the existing guidelines and consensus publications.^73^ In line with our own consensus,^58^ China's diagnosis and treatment guidelines state that EGb 761^®^ is effective in the treatment of AD, multi‐infarct dementia, and MCI.^70^ Outside Asia, Czech consensus guidelines recommend EGb 761^®^ in patients with incident dementia/MCI with a Mini‐Mental State Exam score of >25;^72^ and a Swiss Expert Recommendation includes initiating EGb 761^®^ treatment during the MCI stage.^71^ A Spanish consensus document states that EGb 761^®^ is the only approved drug treatment for MCI.^96^ In addition, the European Medicines Agency (EMA) assessment report (2015) recommends EGb 761^®^ for improvement of (age‐associated) cognitive decline and quality of life in patients with mild dementia.^68^

**FIGURE 1:**
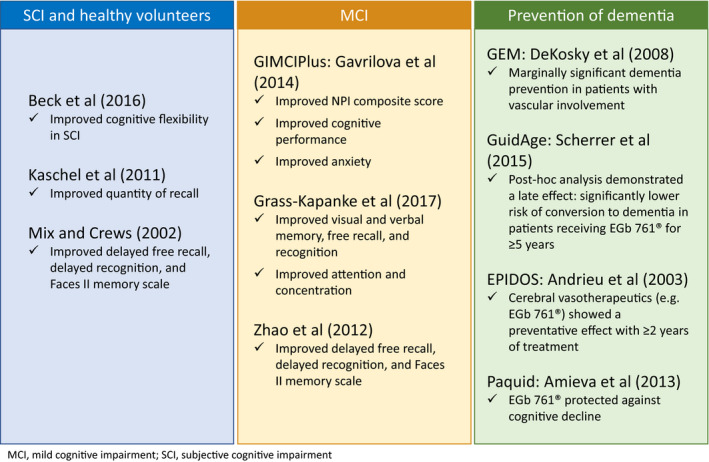
Key trials of EGb 761^®^ indicating cognitive benefits in healthy individuals and MCI patients, and effects on dementia prevention

Four key trials have reported benefit from EGb 761^®^ in patients with MCI (Figure 1 and Table 1). The double‐blind, randomized GIMCIPlus trial^93^ enrolled 160 outpatients aged ≥55 years with amnestic MCI with NPS, diagnosed in accordance with the International Working Group (2004).^24^ This study population also met the DSM‐5 diagnostic criteria for mild neurocognitive disorder when retrospectively applied.^97^ Patients were randomly assigned to receive EGb 761^®^ 240 mg/day or placebo, and NPS were assessed after 12 and 24 weeks of treatment using the 12‐item Neuropsychiatric Inventory (NPI). The Clinical Global Impression (CGI) scale was used for global assessment. EGb 761^®^ significantly improved NPI composite scores versus placebo (mean, –7.0 vs. –5.5, respectively; *p* = 0.001), including a significant improvement in anxiety and a trend toward improved depression. NPI improvement by ≥4 points was achieved by 79% vs. 56% of patients (*p* = 0.002). EGb 761^®^‐treated patients also showed significantly improved cognitive performance assessed by the Trail‐Making Test, particularly visuomotor speed and executive functioning, compared with those receiving placebo.^93^

**Table 1:** Summary of trials of EGb 761^®^ in patients with MCI and healthy subjects, and dementia prevention

Trial	Study design	*N*	Population	Key outcomes
**EGb 761^®^ in patients with MCI**				
GIMCIPlus study Gavrilova et al, 2014^93^	Randomized to EGb 761^®^ (240 mg/day) or placebo for 24 weeks	160	Patients aged ≥55 years with amnestic MCI with NPS	EGb 761^®^ significantly improved NPI composite scores versus placebo (*p* = 0.001). ≥4‐point NPI improvement achieved by 79% vs. 56% of patients (*p* = 0.002). EGb 761^®^ associated with improved cognitive performance, particularly visuomotor speed and executive functioning scores (*p* < 0.05), vs. placebo Significant improvement in anxiety (*p* = 0.027) and a trend toward improved depression (*p* = 0.066) with EGb 761^®^.
Grass‐Kapanke et al, 2011^95^	Randomized to EGb 761^®^ (240 mg/day) or placebo for 12 weeks	300	Patients aged 45–65 years with vMCI	EGb 761^®^ significantly improved visual memory (Faces I, *p* = 0.04), and free recall/recognition under both immediate (*p* = 0.06) and delayed conditions (*p* = 0.03). EGb 761^®^ improved attention (*p* = 0.21) and concentration (*p* = 0.01) outcomes. Cognitive effects were more pronounced and consistent in subjects with lower baseline memory function. Improved perceived physical health (*p* = 0.04) and a trend in favor of improved perceived mental health (*p* = 0.15) with EGb 761^®^.
Zhao et al, 2012^94^	Randomized to EGb 761®^a^ or general healthcare alone for 6 months	120	Patients aged 60–85 years with MCI	Only the EGb 761^®^ treatment group achieved significant improvement from baseline in logical memory and picture recognition. Clinical and logical memory tests were significantly improved with EGb 761^®^ versus health care alone.
**EGb 761^®^ in patients with SCI and cognitively healthy subjects**				
Beck et al, 2016^92^	Randomized to EGb 761^®^ *(*240 mg/day) or placebo for 8 weeks	61	Elderly individuals with subjective memory complaints	Improved cognitive flexibility (task switching, *p* = 0.018) without brain activation changes in patients receiving EGb 761^®^, suggesting improved processing efficiency.
Kaschel et al, 2011^99^	Randomized to EGb 761^®^ (240 mg/day) or placebo for 6 weeks	188	Healthy subjects aged 45–56 years	EGb 761^®^ showed significant improvement in the high‐demand prospective memory task of quantity of recall (number of correctly recalled appointments) compared with those receiving placebo, under both immediate (*p* = 0.038) and delayed conditions (*p* = 0.008). No superiority in the less‐demanding driving route memory test.
Mix and Crews, 2002^98^	Randomized to EGb 761^®^ *(*180 mg/day) or placebo for 6 weeks	262	Healthy subjects aged ≥60 years	EGb 761^®^ significantly improved delayed free recall (*p* < 0.04), delayed recognition (*p* < 0.01), and the Faces II memory scale (*p* < 0.025), compared with placebo.
**EGb 761^®^ for the prevention of dementia**				
GEM study; DeKosky et al, 2008^100^	Randomized to EGb 761^®^ 120 mg BID or placebo. Median follow‐up, 6.1 years	3069	Mean age, 79 years (482 had MCI at baseline; 2587 had normal cognition)	18% of patients receiving EGb 761^®^ and 16% receiving placebo developed dementia (HR, 1.12; 95% CI, 0.94–1.33). HR for the VaD subgroup was marginally significant (HR, 0.41; 95% CI, 0.17–0.98; *p* = 0.05).
GuidAge study; Vellas et al, 2012^101^; Scherrer et al, 2015^102^	Randomized to EGb 761^®^ 240 mg/day or placebo for 5 years	2854	Patients aged >70 years with memory complaints	4% of patients receiving EGb 761^®^ (1.2 cases/100 person‐years) and 5% receiving placebo (1.4 cases/100 person‐years) developed AD (HR, 0.84; 95% CI, 0.60–1.18; *p* = 0.306). Significant preventive benefit in patients receiving EGb 761^®^ for ≥5 years (*p* = 0.034). Statistical testing for late effect showed a significantly lower risk of progression to dementia with EGb 761^®^ versus placebo (*p* = 0.0054)
Andrieu et al, 2003^103^	Paired, case‐control nested study from EPIDOS trial; 7‐year follow‐up	414	Community‐dwelling women aged >75 years: 69 with AD dementia + 345 with normal cognitive function	Among individuals who developed AD dementia, significantly fewer had received EGb 761^®^ or other cerebral/peripheral vasotherapeutics for ≥2 years, vs. those with no AD (*p* = 0.018). For EGb 761^®^ only, trend in favor of AD prevention (*p* = 0.22)
Amieva et al, 2013^105^	Data from prospective PAQUID study (20‐year follow‐up)	3612	Nondemented individuals aged ≥65 years	EGb 761^®^ protected against cognitive (MMSE) decline over 20 years (*p* < 0.0001) versus no treatment. In contrast, piracetam treatment showed poorer cognitive decline vs. no treatment.

Abbreviations: AD, Alzheimer's disease; HR, hazard ratio; MCI, mild cognitive impairment; MMSE, Mini‐Mental State Examination; NPI, Neuropsychiatric Inventory; SCI, subjective cognitive impairment; TID, three times daily; VaD, vascular dementia; vMCI, very mild MCI.

^a^ Dosage was stated as 19.2 mg TID, which is assumed to refer to the dose of flavone glycosides in a therapeutic dose of EGb 761^®^.

Additionally, a 2011 trial published by Grass‐Kapanke et al^95^ included 300 patients aged 45–65 years with very mild MCI, defined as cognitive complaints and low functioning in at least one of the cognitive tests. The patient population met the later‐introduced DSM‐5 diagnostic criteria for mild neurocognitive disorder.^97^ Patients were randomly assigned to receive EGb 761^®^ 240 mg/day or placebo once daily for 12 weeks. Patients receiving EGb 761^®^ showed significant improvement in both visual and prospective verbal memory, free recall, and recognition, under both immediate and delayed conditions. EGb 761^®^ also improved attention and concentration outcomes, and trended in favor of improved facial recognition and perceived physical health. Cognitive benefits were more pronounced among subjects with poorer baseline memory function.^95^

A multicenter trial by Zhao et al^94^ included 120 patients aged 60–85 years with MCI including episodic memory complaints. Patients were randomized 1:1 to receive EGb 761^®^ or general healthcare alone for 6 months. Only the EGb 761^®^ treatment group achieved significant improvement from baseline in logical memory and picture recognition. After 6 months, the clinical memory scale (picture recognition) and logical memory tests were significantly improved from baseline with EGb 761^®^ (*p* < 0.01 and *p* < 0.05, respectively), while health care alone showed no significant improvement in these endpoints. In a comparison of the two approaches, clinical and logical memory were significantly improved with EGb 761^®^ versus health care alone (*p* < 0.05 for both).

Furthermore, a placebo‐controlled study was conducted in 61 elderly individuals with subjective memory impairment. Participants were randomized to receive EGb 761^®^ 240 mg/day or matching placebo for 8 weeks. EGb 761^®^ improved cognitive flexibility without brain activation changes, suggesting improved processing efficiency potentially resulting from mild enhancement of prefrontal dopaminergic function.^92^

### 3.4 Studies of EGb 761^®^ in cognitively healthy volunteers

In addition, two randomized, placebo‐controlled studies have shown encouraging results in healthy subjects (Figure 1 and Table 1). A 2002 study by Mix and Crews reported that EGb 761^®^ 180 mg/day significantly improved delayed free recall (*p* < 0.04), delayed recognition (*p* < 0.01), and the Faces II memory scale (*p* < 0.0025) compared with placebo after 6 weeks of treatment in cognitively intact individuals aged ≥60 years.^98^ A decade later, Kaschel et al^99^ reported that cognitively healthy individuals aged 45–56 years who received EGb 761^®^ showed significant improvement in the high‐demand prospective memory task of “quantity of recall” (ie, the number of correctly recalled appointments) after 6 weeks compared with placebo, both in terms of immediate recall (*p* = 0.038) and delayed recall (*p* = 0.008).

### 3.5 Trials of EGb 761^®^ in delaying dementia

Given that individuals with MCI progress to clinically evident AD at an accelerated rate compared with healthy individuals,^3^, ^6^, ^7^, ^8^, ^9^ diagnosis of MCI has been recognized as an important opportunity to attempt preventative intervention.^2^, ^3^, ^22^

Two multicenter, randomized, double‐blind, placebo‐controlled trials (the Ginkgo Evaluation of Memory [GEM]^100^ and GuidAge^101^ studies) evaluated the use of EGb 761^®^ in the prevention of dementia (Figure 1 and Table 1). While neither study demonstrated a clear effect in prevention of dementia in the initial analyses, both results should be interpreted in the context of the specific study limitations.

In the GEM study, of the 3069 volunteers, 482 (16%) had MCI and 2587 (84%) had normal cognition. At a median follow‐up of 6.1 years, 18% of patients receiving EGb 761^®^ and 16% of those receiving placebo developed dementia. The hazard ratio (HR) for all‐cause dementia was 1.12 (95% CI, 0.94–1.33), and for AD, the HR was 1.16 (95% CI, 0.97–1.39).^100^ However, these data could have been confounded by several factors, including the advanced age of the study population (mean, 79 years), the lower incidence of VaD in the EGb 761^®^ group compared with the placebo group (7 vs. 17 per 100 person‐years; HR, 0.41; *p* = 0.05), and the decreasing level of compliance over the course of the study (only 60% at 6 years). The authors also acknowledged that, given the often lengthy delay from initial brain changes to the diagnosis of clinical dementia, the effect of EGb 761^®^ may take many more years of follow‐up to manifest.^100^

In the randomized, placebo‐controlled GuidAge study, patients aged >70 years with memory complaints received either EGb 761^®^ 240 mg/day or placebo for five years. Sixty‐one of 1406 patients receiving EGb 761^®^ (1.2 cases/100 person‐years) and 73 of 1414 receiving placebo (1.4 cases/100 person‐years) were diagnosed with incident AD during follow‐up (HR, 0.84; 95% CI, 0.60–1.18; *p* = 0.306).^101^ While a significant difference between the groups was not shown in the initial analysis, which applied a test based on the assumption of proportional hazards, the risk of AD was not constant over time. Results from a preplanned subgroup analysis showed significant benefit in those patients exposed to EGb 761^®^ for at least four years (rate of progression to AD, 1.6% vs. 3.0%, respectively; *p* = 0.03). Subsequently, a *post hoc* analysis of the GuidAge study was performed, which demonstrated a possible late effect of EGb 761^®^.^102^ This analysis applied additional statistical testing to explicitly test the hypothesis of a late treatment effect. In a protocol‐specified subgroup analysis, a significant treatment‐by‐time interaction for AD incidence was demonstrated; and a subsequent analysis using the Fleming‐Harrington test for late effect showed a significantly lower risk of progression to dementia with EGb 761^®^ versus placebo (*p* = 0.0054).^102^

Other data have also indicated a possible role for EGb 761^®^ in delaying dementia onset. For example, the EPIDOS nested case‐control study evaluated community‐dwelling women aged >75 years, among whom 69 developed AD dementia, and 345 paired women retained normal cognitive function. Among those that developed dementia, significantly fewer had received EGb 761^®^ or other peripheral vasotherapeutics for at least 2 years (OR, 0.31; 95% CI, 0.12–0.82; *p* = 0.018), compared with the group who did not develop AD dementia, suggesting a possible preventative effect.^103^ The benefits of taking EGb 761^®^ were evident after one year of treatment.^104^

Further, in a retrospective 20‐year follow‐up analysis of the PAQUID study in 3612 nondemented individuals aged ≥65 years, EGb 761^®^ appeared to protect against cognitive decline in the long term. Mini‐Mental State Examination data showed strongly improved cognitive outcomes with EGb 761^®^ versus no treatment (*p* < 0.0001). While the EGb 761^®^ group declined less rapidly than the “no treatment” group, patients who had received piracetam declined more rapidly, suggesting a specific medication effect of EGb 761^®^.^105^

It should be emphasized that not all studies have demonstrated a protective effect with EGb 761^®^,^106^ and the *post hoc* analyses described above were intended to be hypothesis‐generating exercises. The retrospectively applied statistical tests do, however, justify further prospective, well‐controlled, long‐term studies of EGb 761^®^ in patients with early cognitive decline and MCI, to more fully establish the role of this agent in delaying or preventing dementia.

### 3.6 EGb 761^®^ safety

EGb 761^®^ has demonstrated a positive risk‐benefit profile.^52^, ^88^ Studies and meta‐analyses have consistently shown no significant increase in overall risk of adverse events with EGb 761^®^ versus placebo.^52^, ^54^, ^55^, ^56^, ^89^, ^93^, ^95^, ^107^ Indeed, one meta‐analysis showed a numerically lower rate of discontinuation in EGb 761^®^‐treated patients versus those receiving placebo.^89^

Historically, some concern has been raised regarding increased bleeding risk in patients treated with EGb 761^®^. This issue was discussed in detail in our previous consensus publication.^58^ Briefly, the data from published trials and meta‐analyses show no evidence of an increased risk of bleeding with EGb 761^®^,^107^, ^108^ nor any clinically important changes in bleeding time, coagulation parameters, or platelet aggregation in doses up to 480 mg/day.^108^, ^109^, ^110^, ^111^ There also appears to be no additive effect of EGb 761^®^ with aspirin,^112^ and EGb 761^®^ does not change the pharmacokinetic or pharmacodynamic properties of simultaneously administered warfarin.^113^

## 4 CONCLUSIONS AND EXPERT CONSENSUS

Key studies that evaluated EGb 761^®^ in MCI patients, individuals with SCI, and cognitively healthy individuals, are summarized in Table 1. In Table 2, we summarize the ASCEND group's expert consensus recommendations regarding the use of EGb 761^®^ in the management of MCI with or without CVD and NPS, along with other statements on clinically relevant issues. Consensus statements presented herein are primarily based on results from the key randomized trials discussed above, in tandem with clinical expertise.

**Table 2:** Summary of ASCEND expert consensus statements

***Diagnosis of MCI***	
It is recommended that clinicians assess for MCI using validated tools, including testing for functional assessment (ADL).	Level of evidence C (expert opinion)
Biomarker assessments, including imaging, may help confirm a diagnosis of MCI, and establish the presence of CVD (vascular pathology).	Class of recommendation IIa Level of evidence B
As far as possible, we recommend the use of consistent MCI diagnostic criteria in clinical trials, to enable more robust conclusions.	Level of evidence C (expert opinion)
***General management principles***	
It is important to identify MCI patients who are at higher risk for dementia, to ensure early intervention.	Level of evidence C (expert opinion)
Clinicians should first assess for reversible causes of MCI impairment, and treat and follow‐up accordingly.	Level of evidence C (expert opinion)
A multidomain intervention strategy is useful in MCI to benefit both neurodegenerative and vascular pathologies. Such a strategy should at least include physical exercise, smoking cessation, management of hypertension and diabetes, cognitive training, and psychosocial interventions.	Class of recommendation I Level of evidence B
***Symptomatic MCI treatment with EGb 761®***	
There is a lack of robust evidence supporting the use of AChEI in improving MCI symptoms.	Class of recommendation III Level of recommendation A
EGb 761^®^ has demonstrated improvement in MCI symptoms in at least four randomized trials, and is the only pharmacological agent recommended in existing guidelines for the symptomatic treatment of MCI.	Class of recommendation I Level of evidence A
It is clinically appropriate to incorporate EGb 761^®^ as part of the multidomain intervention for MCI.	Class of recommendation IIB Level of evidence A
EGb 761^®^ may improve cognitive performance in MCI patients.	Class of recommendation I Level of evidence A
EGb 761^®^ may improve NPS	Class of recommendation IIB Level of evidence B
Due to its beneficial effects on cerebrovascular blood flow, it is reasonable to expect that EGb 761^®^ may benefit MCI patients with CVD.	Level of evidence C (expert opinion)
***Role of EGb 761^®^ in delaying dementia***	
Given that individuals with MCI progress to clinically evident AD at an accelerated rate compared with healthy individuals, diagnosis of MCI represents an important opportunity for initiating therapy.	Level of evidence C (expert opinion)
Based on *post hoc* evidence from two randomized studies, EGb 761^®^ may help delay progression of MCI to dementia in some individuals.	Class of recommendation IIb Level of evidence C
There is justification for well‐controlled, long‐term prospective studies of EGb 761^®^ in patients with early cognitive decline and MCI, to more fully establish the role of this agent in delaying or preventing dementia.	Level of evidence C (expert opinion)
***EGb 761^®^ Safety in MCI***	
EGb 761^®^ has a favorable risk‐benefit profile.	Level of evidence A
No evidence of an increased risk of bleeding has been demonstrated with EGb 761^®^.	Level of evidence A
No significant interaction of EGb 761^®^ with concomitant anticoagulants or antiplatelet agents has been demonstrated.	Level of evidence B
***KEY:*** ***Class of recommendation*** Class I: Evidence and/or general agreement that a given treatment or procedure is beneficial, useful, effective (is recommended/is indicated) Class IIa: Weight of evidence/opinion is in favor of usefulness/efficacy (is reasonable to consider) Class IIb: Usefulness/efficacy is less well established by evidence/opinion (may be reasonable to consider) Class III: Evidence or general agreement that the given treatment or procedure is not useful/effective, or in some cases may be harmful (is not recommended) ***Level of evidence*** A: Data derived from multiple randomized, placebo‐controlled clinical trials, or meta‐analyses B: Data derived from a single randomized clinical trial or large nonrandomized studies C: Consensus of opinion of experts and/or case reports, small studies, retrospective studies	

Abbreviations: AChEI, acetylcholinesterase inhibitors; ADL, activities of daily living; CVD, cerebrovascular disease; MCI, mild cognitive impairment; NPS, neuropsychiatric symptoms.

Among the available anti‐dementia drugs and supplements, EGb 761^®^ is currently the only agent to have demonstrated positive effects in randomized trials in MCI patients.^58^, ^73^ EGb 761^®^ has been shown to enhance cognitive function and efficiency in patients with clinically diagnosed MCI,^92^, ^93^, ^94^, ^95^ particularly in those with greater severity of disease at baseline.^95^ For amnestic MCI patients with NPS, EGb 761^®^ has been shown to improve cognitive function and NPS.^93^ Furthermore, based on the known effects of EGb 761^®^ in promoting brain circulation, protecting against oxidative stress, and providing neuroprotective effects,^58^ it is rational to expect that EGb 761^®^ may benefit MCI patients with concomitant CVD, but this hypothesis needs to be verified in further clinical trials. This knowledge may be particularly important among Asian populations who appear to have a higher likelihood of vascular involvement.^41^, ^42^

The higher risk of developing dementia after an MCI diagnosis represents a strong argument to initiate treatment at this earlier stage of the disease continuum. The evidence suggests a possible role for EGb 761^®^ in delaying the progression of cognitive decline and reducing the risk of progression to dementia in some patients,^93^, ^95^ but further studies are warranted. The risk of progression from MCI to dementia may be further reduced by eliminating risk factors, including vascular risk factors. Future studies should attempt to clarify the impact of early lifestyle interventions with or without EGb 761^®^.

Additional data are desirable to further inform clinical practice and select appropriate patients for EGb 761^®^ treatment. Longitudinal studies with imaging investigations and biomarker analyses would be helpful, to assess the efficacy of EGb 761^®^ in subgroups of MCI patients with differing underlying pathologies, including those with CVD or amyloid plaques. The role of EGb 761^®^ in MCI patients with mild behavioral impairment is also currently not clarified; nor is there any clear information as to the differential benefit of EGb 761^®^ in amnestic versus nonamnestic MCI. Further targeted research in these areas will be important future pursuits. We were able to locate only one small randomized trial of EGb 761^®^ in subjective memory impairment. Given that individuals with SCI are understood to be at higher risk for future cognitive decline,^114^, ^115^ additional evidence investigating earlier EGb 761^®^ initiation before clinically diagnosable MCI or dementia would be highly informative. In addition, data specific to Asian patients are lacking; replication in Asian populations of RCTs previously conducted in Western populations would provide valuable comparisons between populations.

More information is also needed on how and when to initiate EGb 761^®^ treatment, and for how long. Subanalysis of the existing EGb 761^®^ efficacy data stratified by patient body weight would clarify whether the 240 mg daily dose of EGb 761^®^ is effective independent of weight. Furthermore, longer‐term EGb 761^®^ data will be highly valuable, given that clinically diagnosed MCI is a chronic condition, often with underlying CVD or other pathologies which take time to manifest improvement. The available randomized trials that assessed symptom improvement in MCI or SCI followed patients over only 2–6 months of treatment. Study of EGb 761^®^ efficacy by treatment duration would be a valuable addition to the existing body of data, to help inform clinicians as to the optimum duration of therapy, and whether ongoing improvement, or a plateau in benefit, might be expected over the long term. There is also a lack of multidomain trials evaluating the benefits of combining EGb 761^®^ in combination with nonpharmacological strategies such as cognitive training, physical activity, nutritional interventions, and cardiovascular risk factor management.

Summing up the results presented herein, the ASCEND2 group concluded that, based on the available data, the *Ginkgo biloba* extract, EGb 761^®^ has a role in the multidomain intervention strategy for MCI management. Our recommendations are intended to further contribute to the improvement of clinical practice and patient outcomes within Asia. Healthcare professionals within the region are encouraged to consider these recommendations when formulating appropriate strategies for the treatment and management of MCI, with a view to improving patient outcomes.

## CONFLICT OF INTEREST

All authors participated in the ASCEND expert meeting from which this document was drafted, and contributed to or critically reviewed the manuscript. The final manuscript was approved by all authors prior to publication. The following authors declare no conflict of interest: Yuda Turana, Saedon Nor'izzati, Christopher Chen, Narayanaswamy Venketasubramanian, Young Chul Youn, Harjot Singh, Siti Setiati, SangYun Kim, Seol‐Heui Han, Ralf Ihl, Jianping Jia, Trang Tong Mai, and Fee Mann Yong. During the last three years: Nagaendran Kandiah has received grant funding and honorarium payments from Novartis Pharmaceuticals, Eisai Pharmaceuticals, Lundbeck, Schwabe Pharma. Chan Yee Fai has received honoraria and CME sponsorship from DCH Auriga, Eisai, Johnson & Johnson, Lundbeck and Novartis as well as involvement in Industry Sponsored Research. Encarnita Ampil has received honoraria from the Lundbeck, Menarini, Medichem, Torrent, Novartis, Kusum Pharma for CME sponsorship, lecture fees and/or module development. D. Darwin A. Dasig. MD has received honoraria, CME sponsorship, lecture fees and/or speaker fees from Hi‐Eisai, Lundbeck, Medichem, and Menarini. Ng Li‐Ling has received honoraria from Novartis, Eisai, MIMS, Schwabe Pharma, and Danone Dumex, and was involved as a consultant, speaker, or in advisory boards for Novartis, Eisai, MIMS, Schwabe Pharma, and Danone Dumex. Paulus Anam Ong has received CME lecture fees from Lundbeck Indonesia, and a grant from Universitas Padjadjaran Bandung Indonesia. Jacqueline Dominguez has received honoraria and CME sponsorship from HI‐Eisai Pharmaceutical Phil., Inc, Menarini Inc Philippines, and Schwabe Pharma; was involved as a consultant, speaker, or in advisory boards for HI‐Eisai Pharmaceutical Phil., Inc, and Menarini Inc Philippines, and EVER Pharma; and received a research grant from the Philippine Institute for Traditional and Alternative Healthcare‐Department of Health. Nguyen Dinh Toan has received honoraria from Eisai pharmaceutical company. Panita Limpawattana has received CME sponsorship from A. Menarini (Thailand) Limited. Chuthamanee Suthisisang has received CME sponsorship from A. Menarini (Thailand) Limited. Vorapun Senanarong has received lecture fees from Eisai Thailand, Lundbeck Thailand; was involved as a consultant, speaker, or in advisory boards with Menarini Thailand; and has received a research grant from the Faculty of Medicine in Siriraj Hospital, Thailand Research Fund, and SCB Corporate Social Responsibility (CSR) project.

## Data Availability

The data that support the findings of this study are available from the corresponding author upon reasonable request.
